# Unraveling the conformational landscape of amyloid precursor protein intracellular domain

**DOI:** 10.1016/j.bpj.2025.08.010

**Published:** 2025-08-14

**Authors:** Nabanita Mandal, Marie Skepö

**Affiliations:** 1 Division of Computational Chemistry, Department of Chemistry, Science for Life Laboratory, Lund University, Lund, Sweden; 2 NanoLund, Lund University, Lund, Sweden

## Abstract

The amyloid precursor protein intracellular domain (AICD), a cleavage product of amyloid precursor protein implicated in Alzheimer disease and amyloid lateral sclerosis, is a functionally important but structurally elusive intrinsically disordered protein. In this study, we investigate the conformational ensemble of a 35-residue AICD segment encompassing the conserved YENPTY motif using molecular dynamics simulations, small-angle x-ray scattering, circular dichroism, and nuclear magnetic resonance data. Our results reveal that AICD fluctuates between compact and extended states, exhibiting dynamic secondary structure elements and heterogeneous solvent accessibility. Small-angle x-ray scattering and circular dichroism analyses support a partially compact, flexible ensemble, whereas time-lagged independent component analysis uncovers metastable conformational states. Residue-specific solvent accessibility and hydrogen bonding patterns highlight transient structural stabilization, particularly within the YENPTY motif. These findings highlight the structural plasticity of AICD and offer insights into its potential for interaction and regulatory roles in nuclear signaling and neurodegeneration.

## Significance

The amyloid precursor protein intracellular domain (AICD) is linked to Alzheimer’s disease and ALS, but its intrinsically disordered nature has hindered structural insight. Here, we characterize a 35-residue AICD segment containing the conserved YENPTY motif, essential for protein interactions and signaling. Combining molecular dynamics with SAXS, CD, and NMR data, we show that AICD samples a dynamic ensemble of compact and extended conformations with transient secondary structures. This structural plasticity enables AICD to adapt to multiple binding partners. Motif-specific stabilization and metastable states were identified, offering mechanistic insight into AICD’s role in nuclear signaling and neurodegeneration. These findings provide a framework for therapeutic strategies targeting disordered protein regions in neurodegenerative disease.

## Introduction

Amyloid precursor protein (APP) is a type I transmembrane glycoprotein crucial for the development, maintenance, and function of the central nervous system (1). Initially identified for its role in Alzheimer disease (AD) via the production of amyloid-beta (Aβ) peptides, a key component of amyloid plaques, APP is now recognized as a multifunctional protein involved in synaptic plasticity, axonal transport, neuronal migration, and intracellular signaling (2). Encoded on chromosome 21, APP is widely expressed, with particularly high levels in neurons (3). Structurally, it includes a large extracellular domain, a single transmembrane segment, and a short intracellular C-terminal region (4). Its functions are modulated by posttranslational modifications such as glycosylation, phosphorylation, and proteolytic cleavage. APP is processed via two main pathways: the neuroprotective, nonamyloidogenic pathway and the disease-associated, amyloidogenic pathway (5). In the nonamyloidogenic route, α-secretase cleaves within the Aβ region, preventing Aβ formation and releasing the neuroprotective soluble APP (6). The remaining membrane fragment cleaves by γ-secretase, producing the amyloid precursor protein intracellular domain (AICD) (7). In the amyloidogenic pathway, β-secretase initiates cleavage, followed by γ-secretase, generating Aβ (notably Aβ 42) and AICD (8). Although Aβ has been central to AD research, AICD is increasingly recognized for its regulatory roles in nuclear signaling and gene expression (9).

AICD, typically 50–59 amino acids long, translocates to the nucleus in complexes with adaptor proteins such as FE65 and the transcriptional coactivator Tip60, regulating genes linked to neuronal survival, synaptic function, and Aβ clearance, including neprilysin and GSK-3 β(10). A key functional element of AICD is the YENPTY motif, residues 22–27, a conserved sequence that binds phosphotyrosine-binding domain proteins like FE65, X11/MINT, and Dab1, mediating trafficking, endocytosis, and signaling (11). Interaction with FE65 is essential for AICD’s nuclear entry and transcriptional activity (11). Phosphorylation of residues within this motif can disrupt these interactions, impairing transcription and contributing to neuronal dysfunction (12). Structurally, AICD exhibits characteristics of an intrinsically disordered region, with both flexible and structured elements that enable dynamic interactions with multiple partners (13,14,15). Although prior structural insights have primarily emerged from NMR analyses of AICD in complex with adaptor proteins such as FE65, or from fragment-based peptide modeling, a detailed understanding of the conformational behavior of unbound AICD remains limited. Although a few computational or fragment-based modeling studies have examined aspects of AICD (16) or related APP domains (17), to our knowledge, this study represents the first application of all-atom molecular dynamics (MD) simulations to a biologically active AICD fragment encompassing the conserved YENPTY motif. Our computational results indicate that AICD lacks a stable tertiary structure, instead adopting a dynamic ensemble of conformations consistent with induced fit and conformational selection mechanisms of molecular recognition.

Both AICD and the YENPTY motif have been implicated in neurodegenerative diseases. In AD, aberrant APP processing elevates Aβ levels while impairing AICD-mediated gene regulation (18). In amyloid lateral sclerosis (ALS), AICD accumulation may disrupt axonal transport and mitochondrial function in motor neurons (19). Disrupted AICD signaling, via defective nuclear import or impaired protein interactions, can induce cellular stress and neuronal degeneration (20). To investigate AICD’s structure-function dynamics, we focused on a 35-amino-acid segment encompassing the YENPTY motif and its flanking residues. This region retains critical binding and regulatory sites while simplifying computational analysis. Its composition captures both ordered and disordered features, making it a structurally versatile region of interest in the context of AICD’s potential functional roles, including interactions relevant to transcriptional regulation (21). Although these functions may offer promising therapeutic avenues for central nervous system disorders (22), the present study focuses on the conformational landscape of AICD as a foundation for understanding such mechanisms.

## Materials and methods

### Molecular dynamics simulations

Atomistic MD simulations were conducted using the GROMACS package (version 2023) (23) to study a single AICD peptide chain in water with 10 mM NaCl. The AMBER99SB-ILDN (24) force field was employed along with the TIP4P-D water model (25). The initial configuration of AICD was a linear peptide chain, constructed using Avogadro (version 1.2.0) (26), with charged termini, neutral histidine residues, and side chains modeled at neutral pH, resulting in a net charge of −2e. A dodecahedral simulation box with periodic boundary conditions in all directions was used, ensuring that all atoms were at least 1 nm from the edges. Solvent molecules were replaced with NaCl ions to achieve the desired salt concentration and maintain system neutrality. GROMACS’ leapfrog integration algorithm was used with a time step of 2.0 fs, and the Verlet cutoff scheme handled nonbonded and short-range interactions with a cutoff of 12 Å. Dispersion corrections were applied to energy and pressure. In contrast, the long-ranged electrostatic interactions were computed using the particle mesh Ewald (27) method with cubic interpolation and a grid spacing of 1.6 Å. Bonds involving hydrogen were constrained using the LINCS algorithm (28). The Nosé-Hoover thermostat (29) maintained the temperature at 298 K with a fluctuation time of 1.0 ps, applying a separate coupling group for the peptide. Pressure was controlled isotropically via the Parrinello-Rahman barostat (30) with a coupling time constant of 5.0 ps and a compressibility of $4.5\times 10^{-5}\;{\text{bar}}^{-1}$. Energy minimization was carried out using the steepest descent algorithm, followed by a three-step equilibration process: 0.5 ns in the NVT ensemble (constant number of particles, volume, and temperature), 0.5 ns in the NPT ensemble (constant number of particles, pressure, and temperature), and 1.0 ns in the NPT ensemble, with position restraints applied to the peptide throughout. Five independent simulations were performed, each with a production run of 1 μ s, resulting in a total simulation time of 5 μ s.

### Trajectory analysis

The first 300 ns of each simulation trial were discarded to minimize the effects of initial folding from the straight-chain configuration. The resulting trial trajectories were concatenated using GROMACS’ gmx trjcat tool (23). This process generated a concatenated trajectory consisting of 350,000 frames (23). Using an in-house Python script in combination with the GROMACS gmx gyrate command, the frames were sorted based on the radius of gyration $\left(R_{g}\right)$. This sorting created distinct $R_{g}$ groups, each containing varying numbers of frames. Root mean-square deviation (RMSD) was calculated using the gmx rms module of GROMACS (23). The corresponding kernel density estimate (KDE) plot was generated using the kdeplot function from the Seaborn library in Python, providing a smooth, continuous approximation of the probability density distribution of RMSD values throughout the simulation trajectory. The Python script mimics GROMACS frame index files, which can be employed to generate individual trajectory files for each $R_{g}$ group using the gmx trjconv command. These trajectory files were then used for further analysis. Pair distances, Ramachandran plots, and hydrogen bonding were analyzed with GROMACS’ pairdist, rama, and hbond tools (23). The Ramachandran data were visualized as a 2D histogram, which allowed for the integration of the fraction of total data points within specific plot regions. Clustering was performed using the gmx cluster command with the GROMOS23 method and a cutoff distance of 0.9 nm. Scattering profiles for each of the 350,000 frames were calculated using CRYSOL version 3.2.1 (31), with the hydration shell contrast set to zero and all other parameters at their default values. The previously mentioned in-house script was then used to generate average scattering profiles for each identified $R_{g}$ group and all frames combined. The intraparticle distance distribution function, P(r), was calculated for each average scattering profile using PRIMUS version 3.2.1 (32). Time-lagged independent component analysis (tICA) was performed using Deeptime (33), which allowed the construction of free energy and solvent-accessible surface area (SASA) landscapes with the help of the Pyemma (34) and MDTraj (35) packages, respectively. Uniform Manifold Approximation and Projection (UMAP) was used using a Python package, umap-learn (36). The $\chi^{2}$ value was computed to compare experimental data with simulation results using Eq. 1, where N represents the number of data points, $E_{i}$ is the experimental data point, and $S_{i}$ is the simulated data point (37).

(1) $$\chi^{2}=\sum_{i=1}^{N}\frac{{\left(E_{i}-S_{i}\right)}^{2}}{S_{i}^{2}}$$

To evaluate the accuracy of backbone dihedral angle sampling in the MD simulations, scalar J-coupling constants were calculated as structural observables. Specifically, the JHN−Hα3 and JN−Cα2 couplings sensitive to the ϕ and ψ backbone dihedral angles, respectively, were computed using Karplus-type equations:

(2) JHN−Hα3=Acos2(ϕ)+Bcos(ϕ)+C

(3) JN−Cα2=A′cos2(ψ)+B′cos(ψ)+C′

Dihedral angles were extracted from the equilibrated portions of the simulation trajectories. J-coupling values were computed for each frame and residue, and ensemble-averaged values along with standard deviations were obtained to assess residue-specific conformational variability. For ${}^{3}J_{\mathrm{HN}-\mathrm{H\alpha}}$, the Karplus parameters used were A=6.4, B=−1.4, and C=1.9; for ${}^{2}J_{N-\mathrm{C\alpha}}$, the parameters were $A^{\prime }=2.0$, $B^{\prime }=-0.5$, and $C^{\prime }=0.5$, based on literature-validated empirical relationships (38).

### Small-angle x-ray scattering measurements

The synthesized peptide powder (purity 95%) was obtained from Chemtronica, Stockholm. The peptide was dissolved in a buffer containing 10 mM NaCl and 20 mM Tris (pH 7.5), followed by purification through centrifugal dialysis using Vivaspin 2 centrifugal concentrators with a 2000-molecular weight cutoff membrane. Peptide concentration was adjusted to 0.5 mg/mL before small-angle x-ray scattering (SAXS) measurements. SAXS measurements were performed at the BM29 beamline of the European Synchrotron Radiation Facility, Grenoble, France. Data were collected at a beam energy of 12.5 keV and a temperature of 20°C, covering a momentum transfer range (*q*-range) of 0.00776–0.495 Å^−1^. For each sample, 10 consecutive frames were acquired. Frames exhibiting signs of radiation damage were excluded before averaging (39). Buffer measurements were acquired 10 times before and after each sample measurement and processed in the same manner as the sample data. Background subtraction was performed by subtracting the averaged buffer scattering profile from the averaged sample profile. Logarithmic re-binning of the scattering curves to 100 data points was done using DATREGRID, part of the ATSAS software package (32). Subsequent data analysis was conducted using PRIMUS, also from the ATSAS suite (32). Peptide concentrations were determined using a Nanodrop 2000 spectrophotometer.

### Circular dichroism

The synthesized peptide powder, with a purity of 95%, was procured from Chemtronica, Stockholm. It was dissolved in a buffer composed of 10 mM NaF and 10 mM sodium phosphate (dibasic/monobasic) at pH 7.0 and subsequently purified using Vivaspin 2 centrifugal concentrators equipped with a 2000-Da molecular weight cutoff membrane through centrifugal dialysis. Circular dichroism (CD) spectroscopy, which measures the differential absorption of left- and right-circularly polarized light, was used to assess the secondary structure of the peptide. For CD measurements, the peptide concentration was adjusted to 0.1 mg/mL. The experimental CD spectrum was acquired at 25°C. The resulting ellipticity values were converted to molar circular dichroism (Δε) for further analysis.

(4) $$\Delta \varepsilon \;\left(M^{-1}{\text{cm}}^{-1}\right)=\frac{\text{CD}\;\left(\text{mdeg}\right)\times \text{MRW}\;\left(\text{Da}\right)}{32980\times l\;\left(\text{cm}\right)\times c\;\left(g/L\right)}\text{,}$$

where CD is the measured CD signal, l is the pathlength of the cell, *c* is the protein concentration of the sample, and MRW is the mean residue weight. The MRW is calculated from Eq. 4:

(5) $$\text{MRW}=\frac{\text{MW}}{\text{Sequence}\;\text{length}-1}\text{,}$$

where MW is the molecular weight of the protein.

## Results

### Amyloid precursor protein intracellular domain

**Figure 1:**
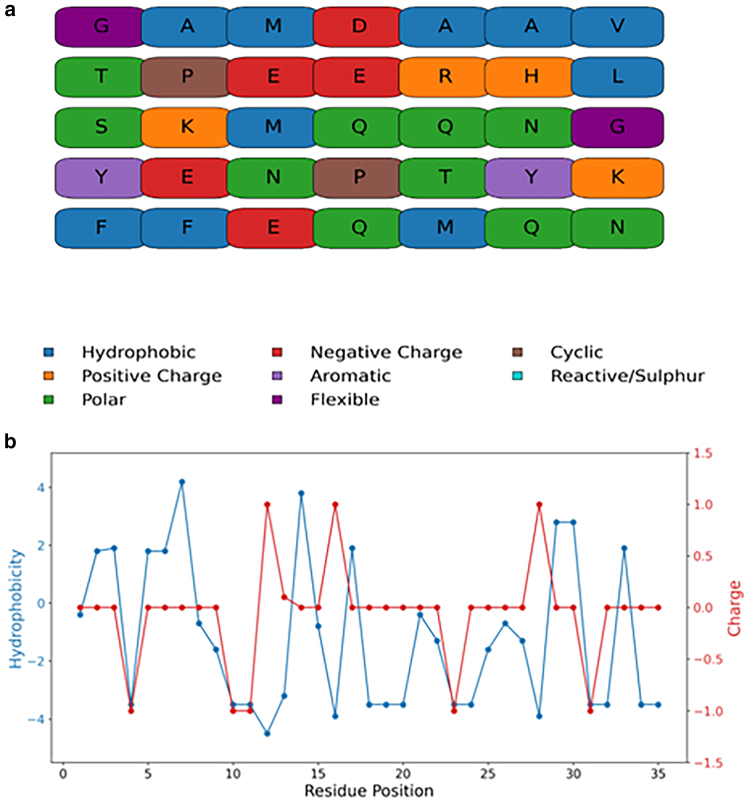
(*a*) Sequence of amyloid precursor protein intracellular domain (AICD) with different color codes depending on the residue property. The YENPTY motif corresponds to residues 22–27. The color code is given in the Figure. Note: Although histidine residues are shown as positively charged for illustrative purposes, in all simulations and structural analyses conducted in this study, histidine was modeled in its neutral form consistent with physiological pH. (*b*) Hydrophobicity (*blue line, left y-axis*) and net charge (*red line, right y-axis*) are shown as functions of residue position of the primary sequence of AICD.

Fig. 1 *a* illustrates the amino acid sequence of the AICD, encompassing residues 1 to 35, with each residue color-coded according to its physicochemical properties. The amino acid sequence exhibits a heterogeneous distribution of hydrophobic, polar, and charged residues, lacking compartmentalized clustering. Hydrophobic residues are scattered throughout the primary sequence, with slight enrichment at the termini, but they do not form a contiguous core. Charged residues are similarly dispersed, with negative charges more prevalent in the N-terminal and central regions.

Fig. 1 *b* illustrates the residue-wise variation in hydrophobicity (blue line, left y-axis) and net charge (red line, right y-axis) along the protein’s primary sequence. Hydrophobicity values were assigned based on the Kyte-Doolittle scale, where positive values represent hydrophobic residues and negative values indicate hydrophilic residues. Charge values were calculated considering the ionization state of each residue at physiological pH. The data reveal alternating regions of hydrophobic and hydrophilic character, suggesting the potential for amphipathic structural motifs. Additionally, positively and negatively charged residues are interspersed along the sequence, with distinct clusters that may contribute to electrostatic interactions or influence the protein’s solubility and folding behavior.

**Figure 2:**
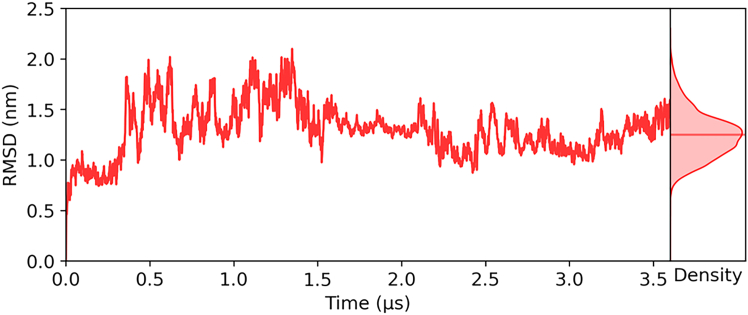
Root mean-square deviation (RMSD) of atomic positions throughout the molecular dynamics simulation, plotted as a function of simulation time (in microseconds).

The structural stability of AICD is assessed by RMSD of atomic positions throughout an MD simulation; see Fig. 2. The RMSD progressively stabilizes as a function of time, with reduced amplitude variations observed beyond 1.5 μ s. This transition reflects the system’s convergence toward a more stable conformational ensemble. A KDE of the RMSD is shown adjacent to the time series plot to further characterize the distribution of sampled conformations. The KDE plot reveals a unimodal distribution centered around approximately 1.2 nm, corresponding to the most frequently sampled deviation from the reference structure. The features of the peak indicate that the majority of the trajectory is dominated by a relatively narrow conformational space, reinforcing the observation of structural stabilization over time. Together, these data suggest that the system has reached a quasi-equilibrium state.

### Structural information obtained through small-angle x-ray scattering and molecular dynamics simulations

**Figure 3:**
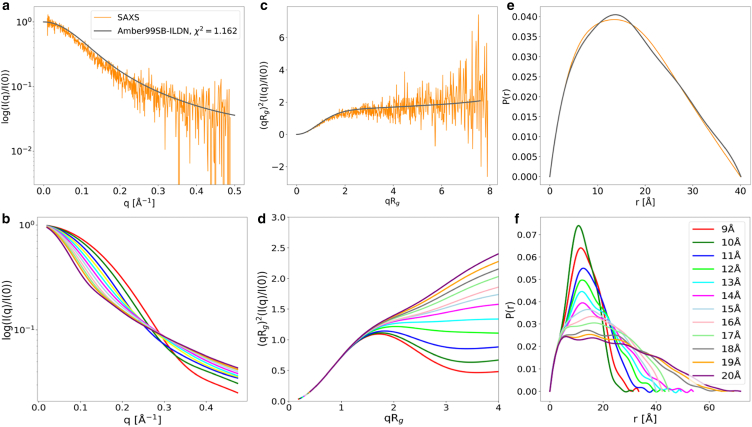
Top row (*a, c,* and *e*): experimental SAXS profile (*orange*) and best-fit model from Amber99SB-ILDN ensemble (*black*), with $\chi^{2}$ fit quality. (*a*) shows the SAXS intensity profile, (*c*) presents the Kratky plot, and (*e*) shows the pair-distance distribution function P(r). Bottom row (*b, d,* and *f*): SAXS intensity, Kratky plots, and P(r) functions from theoretical models with increasing $R_{g}$ (9–20 Å), illustrating how structural expansion influences SAXS profiles and validating the agreement of the Amber99SB-ILDN model with experimental data.

A comparison between experimental and simulated SAXS profiles of AICD is shown in Fig. 3. Experimental data (orange) are compared with simulations over 350,000 frames generated using AMBER99SB-ILDN (dim gray) force fields (40). The experimentally determined average radius of gyration $\left(R_{g}\right)$ is 16.12 Å. In comparison, simulations using the AMBER99SB-ILDN force field yielded average $R_{g}$ values of 16.10 Å and 15.10 Å, respectively. In Fig. 3 *a*, the normalized scattering intensity, log(I(q)/I(0)), is plotted against the scattering vector q. The simulations reproduce the experimental trend well, with a $\chi^{2}$ value of 1.162. The Kratky plot in Fig. 3 *c*, ${\left(qR_{g}\right)}^{2}\left(I\left(q\right)/I\left(0\right)\right)$, demonstrates profiles characteristic of partial disorder. However, the less pronounced elevation at high $qR_{g}$ values suggests that the simulations slightly constrain the extent of conformational fluctuations observed in solution. The P(r) shown in Fig. 3 *e* provides information about the intrapeptide distance distribution. The experimental and simulated P(r)s display a broad peak and an extended tail, indicative of a highly flexible, elongated structure, where the most extended conformation reaches 40 Å. Fig. 3 *b*–*d* and *f* explore different AICD subpopulations, categorized by $R_{g}$ groups. Fig. 3 *b* and *d* depict systematic shifts in the decomposed scattering curves, indicative of a broad distribution of conformers within the conformational ensemble, both with respect to extension and shape. In the latter, there is a variation of shape from relatively extended rods, via disordered, to globular coils. These trends highlight the intrinsic heterogeneity within the AICD ensemble. Regarding decomposition analysis, the $R_{g}$ groups of 10 Å, 15 Å, and 20 Å were selected to represent the conformational ensemble based on their proximity to and distribution around the average $R_{g}$ value of 16.12 Å (1.612 nm) observed during the simulations shown in Figs. S2 and S3. These values allow us to sample conformations from more compact to more extended structures.

### The distribution of solvent-accessible surface area of the decomposed conformational ensemble

**Figure 4:**
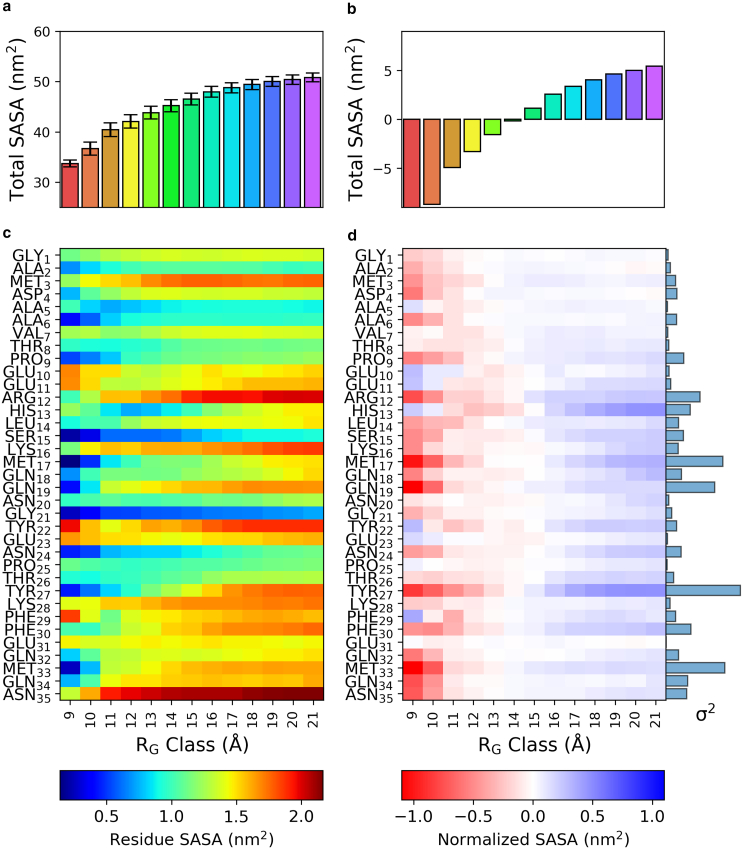
Solvent-accessible surface area (SASA) as a function of structural expansion $R_{g}$: (*a*) bar plot of SASA for different $R_{g}$ groups with error bars representing the standard deviation; (*b*) SASA values normalized by subtracting the mean SASA across all $R_{g}$ groups $\left(∼45\;{\text{nm}}^{2}\right)$ to highlight relative deviations from the ensemble average. This highlights the subtle shifts in solvent exposure that occur during structural expansion: (*c*) nonnormalized and (*d*) normalized per-residue SASA values, along with the corresponding variance detected for each residue, shown as a bar chart in blue.

The conformational fluctuations influencing the exposure of the AICD polypeptide to solvent were analyzed using SASA. Fig. 4 summarizes this relationship using global and residue-specific metrics across distinct $R_{g}$ groups. In Fig. 4 *a*, the total SASA is plotted as a function of $R_{g}$, revealing a monotonic increase, which reflects a transition from more compact states to increasingly extended conformations, wherein a larger fraction of the polypeptide surface becomes solvent exposed. By normalizing SASA with respect to the global mean (Fig. 4 *b*), we unveil an apparent structural dichotomy in AICD conformations: ensembles with smaller $R_{g}$ consistently reside below the mean SASA, reflecting transiently compact states, whereas larger $R_{g}$ ensembles exhibit markedly increased solvent exposure, characteristic of extended conformations. Far from being a trivial observation, this differential pattern underscores AICD’s dynamic equilibrium between compressed and expanded states, which is fundamentally important for its solvent accessibility and interaction potential. Such structural plasticity may be critical in modulating binding interfaces, conformational switching, or functional responsiveness under physiological conditions.

Fig. 4 *c* shows a heatmap of SASA values per residue to further dissect these observations at the residue level. The N-terminal residues G1, A2, and M3 and the C-terminal residues Q34 and N35 display consistently high SASA values, particularly in higher $R_{g}$ groups. These residues are likely to represent disordered, flexible termini that remain solvent exposed across different conformational states. On the other hand, residues such as V7, H13, S15, and M17 show less variation in SASA, indicating their potential role in forming a transiently stabilized hydrophobic core. Interestingly, residues Y22 and F30 show local increases in SASA for higher $R_{g}$, possibly reflecting the transient exposure of hydrophobic side chains during unfolding events.

Fig. 4 *d* further examines the normalized SASA per residue, with color coding to distinguish more exposed residues (blue) or less exposed (red) than their average values across all $R_{g}$ groups. This view identifies dynamic regions of the protein that undergo large conformational changes. Notably, F30 and M33 exhibit red shading in compact structures, suggesting transient burial that may contribute to stabilizing folded-like intermediates. In contrast, F29 does not show this trend consistently, highlighting the heterogeneous nature of residue-specific solvent accessibility in compact ensembles. Q34 and N35 exhibit red shading in compact ensembles, indicating relatively high local solvent accessibility, which gradually shifts to blue in more extended states, suggesting an increase in burial in these conformations.

Importantly, the accompanying bar plot in Fig. 4 *d* quantifies the per-residue variance $\sigma^{2}$ in SASA across the conformational ensemble. Among these, M33 exhibits notably high SASA variance, suggesting substantial fluctuations in its solvent exposure across different conformational states. Such dynamic behavior supports conditionally regulated interactions with protein partners and enzymes involved in posttranslational modifications. The hydrophobic residues F30 and M33 are transiently buried in compact states, potentially stabilizing metastable motifs, whereas their exposure in extended conformations may facilitate molecular recognition. Interestingly, residue Y27 also displays a high SASA variance, as evident from the bar plot, although it was not emphasized in the original discussion. This may indicate that Y27 undergoes notable changes in solvent exposure that are not necessarily linked to structural stabilization or defined interaction sites. Our results indicate that AICD adopts a dynamic ensemble, fluctuating between compact and extended conformations. Solvent accessibility varies across the sequence, with the flexible N- and C-termini generally remaining exposed, whereas residues in the central region exhibit transient compaction.

### Folding and transient structures

**Figure 5:**
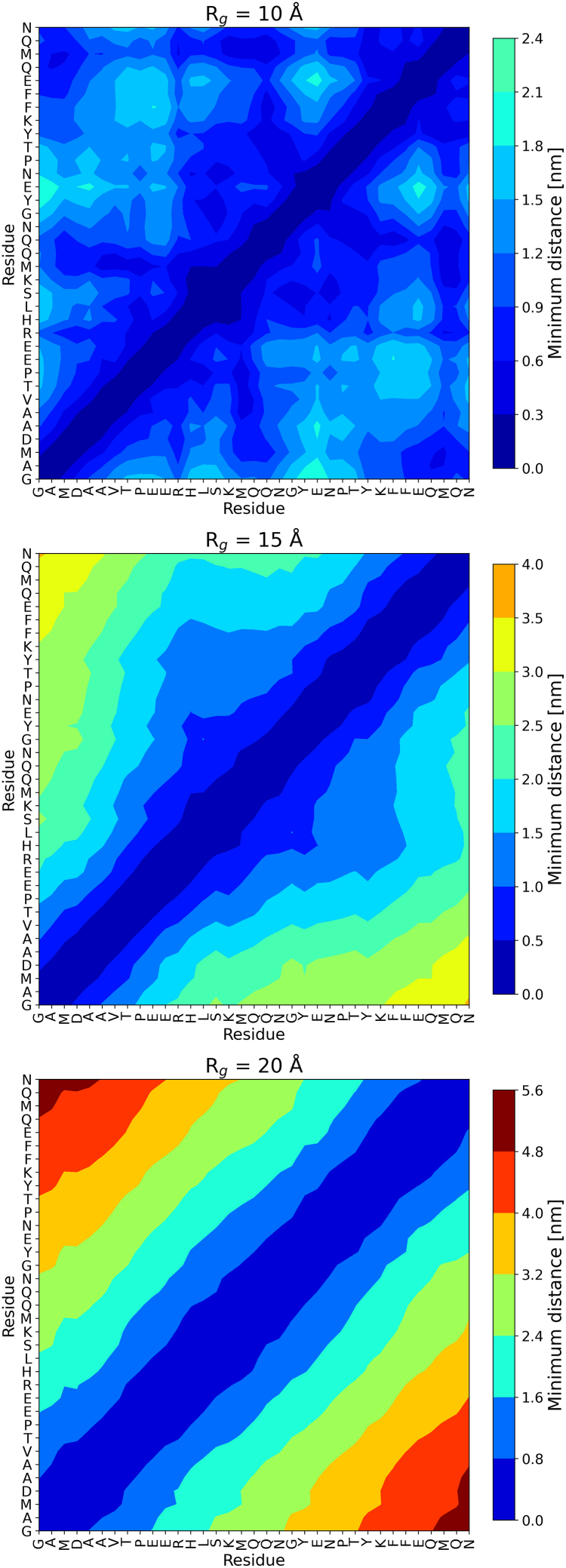
Minimal $C_{\alpha }-C_{\alpha }$ distances contact map of three radii of gyration $\left(R_{g}\right)$ groups sampled by AMBER99SB-ILDN with 10 mM salt concentration.

Contact maps of AICD across the three distinct $R_{g}$ groups are shown in Fig. 5. A trend emerges as a function of $R_{g}$, where lower $R_{g}$ groups exhibit a dense pattern of short-range contacts, highlighted by the prevalence of yellow regions, indicative of tightly packed residue interactions. At higher $R_{g}$ values, close-range contacts diminish significantly, accompanied by a broader distribution of minimal distances across the contact map, indicative of more extended conformations. This observation reflects increased structural flexibility or partial unfolding of AICD, which aligns with the above results.

**Figure 6:**
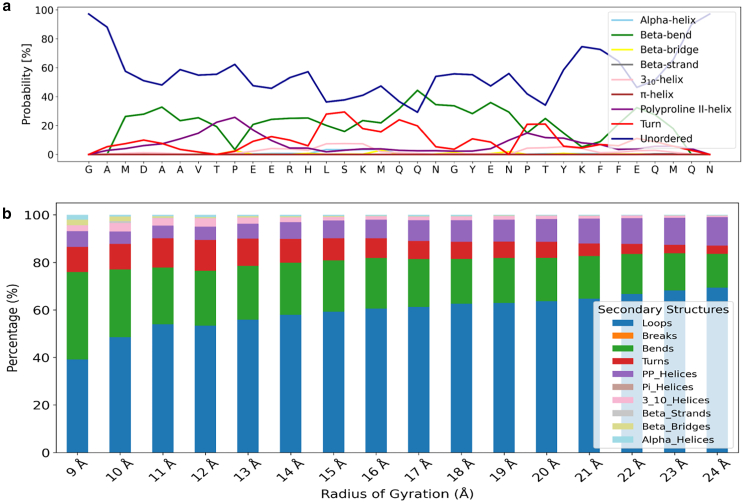
(*a*) Probability of secondary structure occurrence at each residue position in AICD, with individual secondary structure types represented by distinct colors. (*b*) Secondary structure distribution for each radius of gyration $\left(R_{g}\right)$ group, visualized as a stacked bar chart with different secondary structures indicated by color-coding.

Fig. 6 *a* illustrates the residue-level secondary structure probability distribution of the AICD peptide, highlighting its predominantly disordered nature. A significant portion of the sequence exhibits a high propensity for remaining in an unordered state (dark blue), particularly concentrated at the N-terminal and C-terminal regions. Specifically, residues such as G, A, M, D, and V at the N-terminus and K, Q, M, and N at the C-terminus exhibit disorder probabilities exceeding 60%. In contrast, the occurrence of ordered structural motifs such as α-helices (sky blue) and β-strands (gray) is limited overall, with only low-probability and transient elements suggested at scattered positions. Weak α-helical propensities are occasionally observed near residues such as H, L, and K, whereas potential β-strand-like features may occur in segments containing E and P, although these signals remain subtle. In contrast, ordered structural motifs such as α-helices (sky blue) and β-strands (gray) are limited, with only weak and transient tendencies observed at isolated positions. Although low-probability α-helical preferences may be suggested near residues such as H, L, and K, and occasional β-strand-like features appear in segments containing E and P, these patterns are subtle and should be interpreted cautiously. Additionally, intermediate elements like β-bends (green) and turns (red) are more prominently and intermittently distributed along the sequence, with moderate probabilities near residues such as S, Y, E, and Q. These features may act as flexible hinge points that enable localized structural rearrangements within the dynamic ensemble. Fig. 6 *b* further explores the structural distribution of AICD across different $R_{g}$ groups. The analysis reveals that loops (blue), indicative of disordered regions, predominate the structural content across all $R_{g}$ groups, with an increase in loop content as the structure transitions from a compact to an extended state. In addition, bends (green) and turns (red) are also prevalent, reflecting the flexibility of AICD’s structure. The relatively stable presence of more ordered elements, such as α-helices and β-strands, suggests that localized structural features may persist even as AICD adopts more extended conformations.

**Figure 7:**
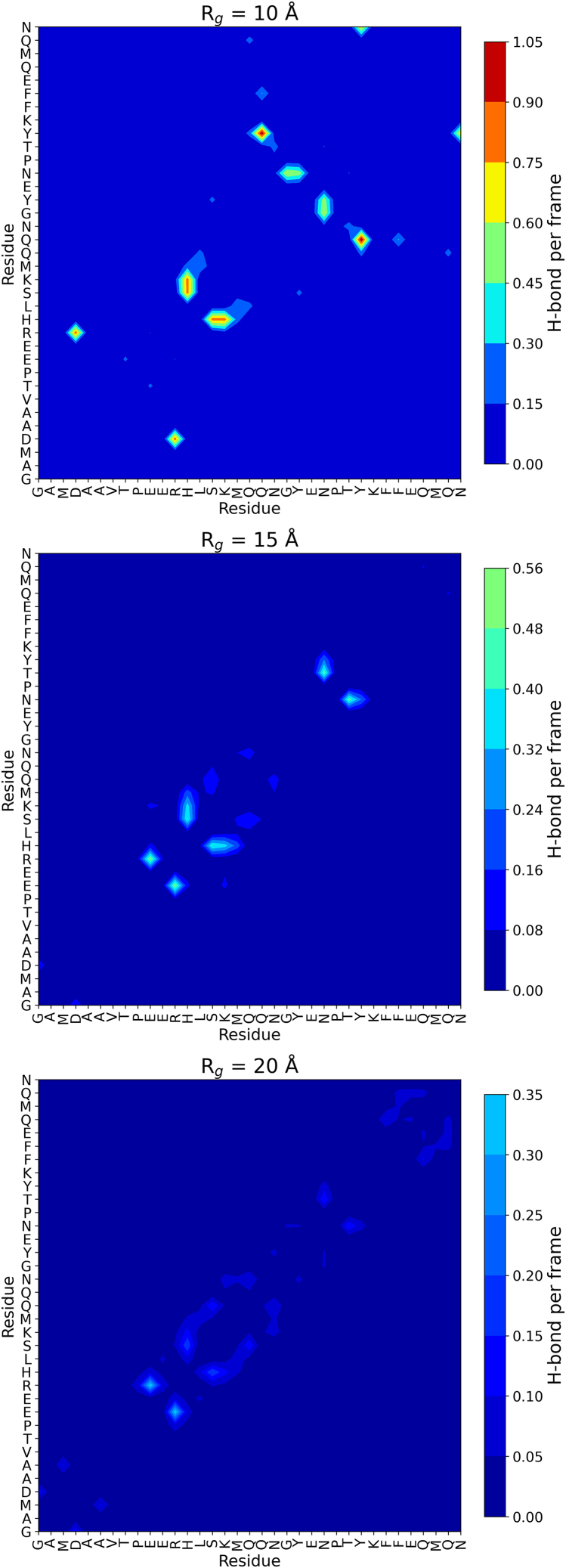
Intramolecular hydrogen bond contact maps at different radii of gyration $\left(R_{g}\right)$ groups.

The contact maps concerning hydrogen bonding in Fig. 7 further confirm our results. At $R_{g}$ = 10 Å, we observe a dense network of intramolecular hydrogen bonds, particularly centered around residues YENPTY (Y20–Y25). These residues exhibit strong interactions within the motif and with nearby segments, suggesting that the motif adopts a semistructured conformation stabilized by local hydrogen bonds. Notably, interactions involving E21, N22, and T24 become prominent in less compact conformers—likely contributing to transient secondary structure or loop formation in the ensemble of structures with $R_{g}\geq$15Å. As the structure expands to $R_{g}$ = 15 Å, the overall hydrogen bond density decreases, and the YENPTY motif shows reduced internal connectivity. However, some interactions persist, particularly involving E21 and T24, indicating that parts of the motif retain transient order even in intermediate states. At $R_{g}$ = 20 Å, representing a highly extended conformation, the hydrogen bonding within the YENPTY motif is markedly diminished. The results suggest that the structural integrity of the YENPTY motif is highly dependent on global compaction, and its ability to form stabilizing intramolecular interactions is lost under extended conditions.

**Figure 8:**
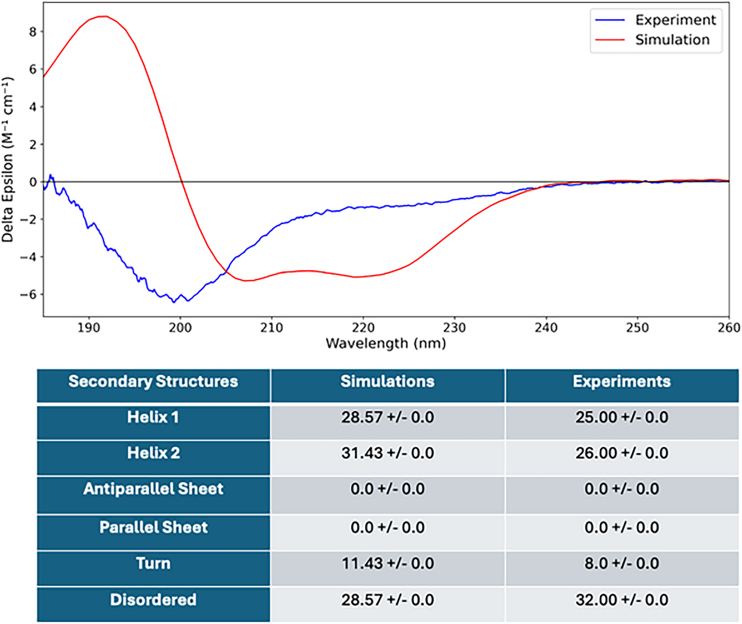
Experimental (*blue*) and simulated (*red*) circular dichroism (CD) spectra of the protein, plotted as molar ellipticity (Δε) versus wavelength. Secondary structure analysis using BeStSel indicates the probability of secondary structure in the protein.

Fig. 8 displays a CD spectrum comparing experimental (blue line) and simulated (red line) data for a protein sample, characterizing its secondary structure composition. The y-axis shows molar ellipticity (Δε), and the x-axis spans wavelengths from 180 to 260 nm. The experimental spectrum features two prominent negative bands near 208 nm and 222 nm, characteristic of α-helical structures. The simulated CD spectrum was calculated by averaging over 350,000 conformations sampled from an MD trajectory. This ensemble based approach captures several general features of the experimental spectrum, although some differences in intensity and spectral shape remain. These discrepancies likely reflect limitations in conformational sampling and the inherent challenge of directly comparing ensemble-averaged experimental data with spectra derived from finite-length simulations. Below the plot, a table summarizes secondary structure content determined using the BeStSel tool (41). The protein comprises mainly helical structures (Helix 1: 28.57%, Helix 2: 31.43%), with no detected β-sheet content. Additionally, the structure includes 11.43% turns and 28.57% disordered regions, indicating a significant level of structural flexibility or intrinsic disorder. Although the spectral match is not perfect, the agreement in secondary structure composition suggests that the simulation provides a reasonable representation of the protein’s structural ensemble. Temperature-dependent CD experiments were not performed in this study; however, such measurements could offer valuable insight into conformational heterogeneity or the presence of multiple structural states. Future studies incorporating temperature variation may help clarify the observed differences between the experimental and simulated spectra.

**Figure 9:**
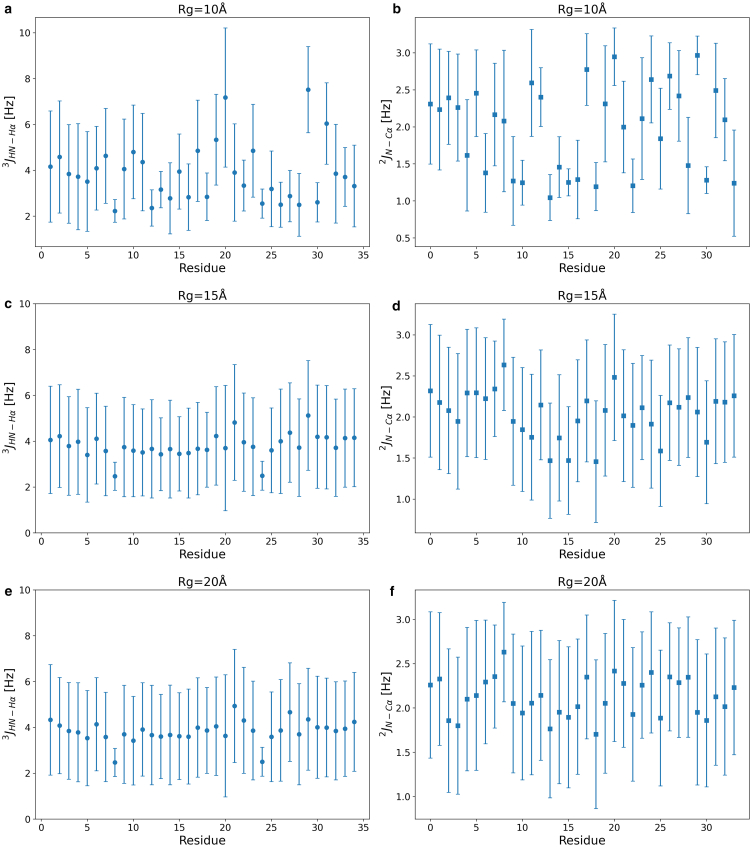
(*a, c,* and *e*) J-coupling for the ϕ (Phi) angles and (*b, d,* and *f*) for the ψ (Psi) angles as a function of residue position. Error bars represent standard deviations, with each marker indicating the mean per residue.

Fig. 9 shows simulation-derived residue-specific scalar coupling constants for three $R_{g}$-defined ensembles (10 Å, 15 Å, and 20 Å), providing insight into local backbone conformations across different levels of compaction. Fig. 9 *a*, *c*, and *e* display JHN−Hα3 values, which are sensitive to backbone ϕ angles, whereas Fig. 9 *b*, *d*, and *f* show JN−Cα2 values, which reflect ψ angle variability. The coupling constants were calculated from MD trajectories using Karplus equations, and the error bars represent the standard deviation of each coupling across the sampled frames, capturing local conformational flexibility.

In the most compact ensemble ($R_{g}=10$ Å), JHN−Hα3 values exhibit pronounced residue-specific variation, suggesting a mixture of α-helical and extended conformations. JN−Cα2 values in this state show moderate variation, indicating some preservation of backbone order. As the ensemble expands ($R_{g}=15$ Å and 20 Å), both types of couplings flatten, and the associated error bars increase, reflecting increased torsional disorder and enhanced conformational heterogeneity. This trend is consistent with the progressive loss of defined secondary structure and the emergence of a more disordered, flexible ensemble. Although these J couplings are not experimental measurements, they serve as sensitive indicators of dihedral angle sampling quality in simulation. Their behavior across compaction states reinforces the interpretation that the system transitions from partially structured to largely disordered conformations.

### The conformational landscape from a time-lagged independent component analysis perspective

**Figure 10:**
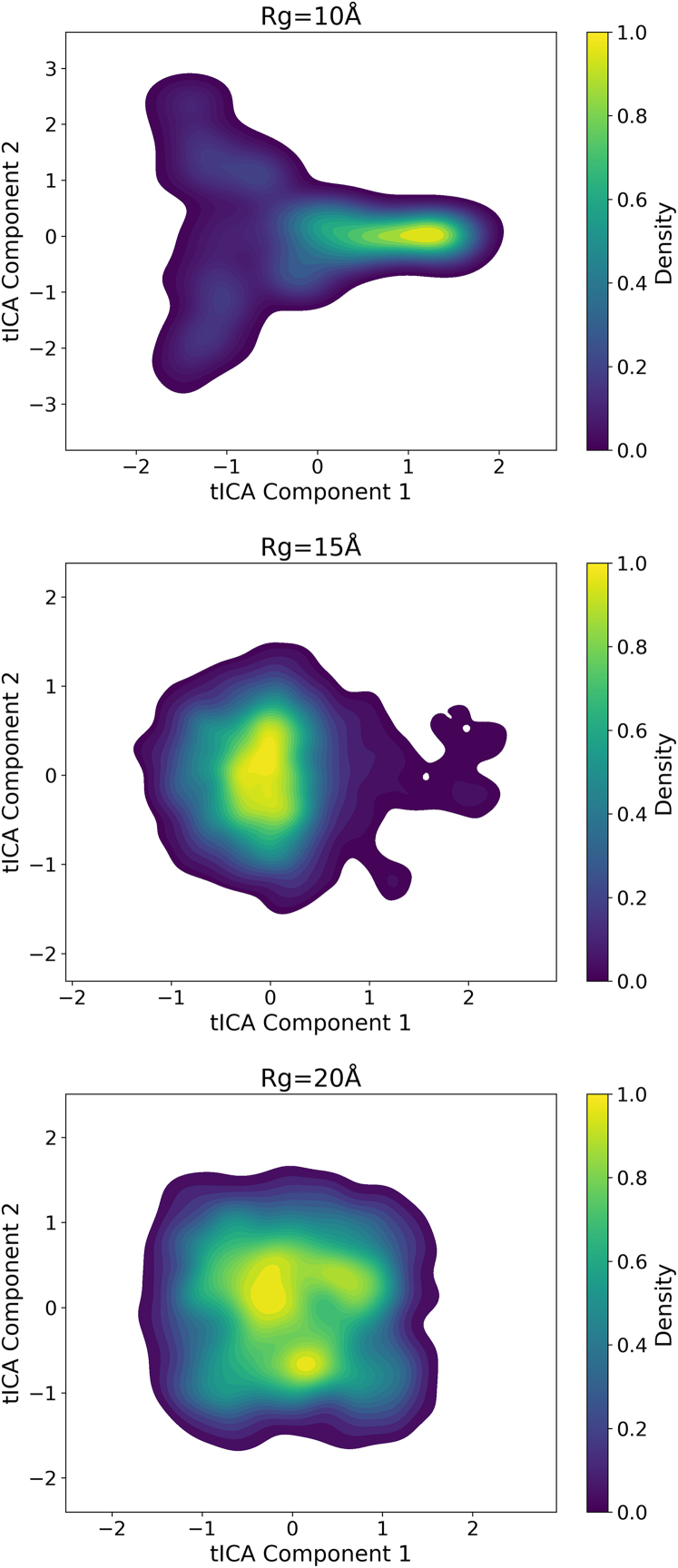
Time-lagged independent component analysis (tICA) performed for the three representative radii of gyration $\left(R_{g}\right)$ groups. The color scale indicating the density is shown beside each plot.

The conformational landscape of AICD projected onto the first two tICA components is given in Fig. 10. tICA identifies the slowest dynamic processes, which could be linked to biologically significant transitions. The distribution of AICD structures reveals multiple metastable states, indicating that AICD does not sample its conformational space uniformly but instead populates distinct regions for prolonged periods before transitioning to other states. We hypothesize that these slow transitions could correspond to functionally critical structural rearrangements. The data for the $R_{g}$ groups provide an additional layer of structural interpretation. The gradual change across tICA space suggests that the expansion and compaction of AICD are slow processes governed by its underlying energy landscape.

### Cluster analysis

**Figure 11:**
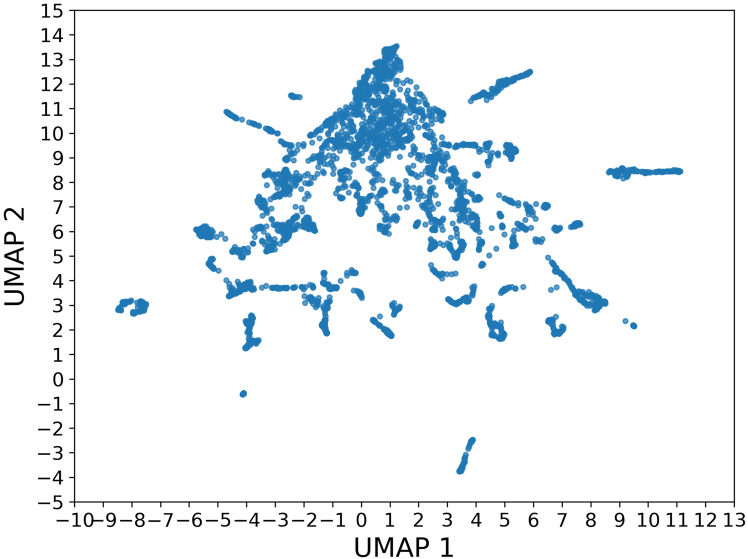
Uniform Manifold Approximation and Projection (UMAP) plot depicting the pairwise distribution of data points, providing a visual representation of their relationships in a reduced-dimensional space.

An integrated visualization of the conformational landscape of AICD, using UMAP analyses, is shown in Fig. 11. The UMAP plot reveals discrete clusters interspersed with more diffuse regions, indicating a mixture of metastable conformations and continuous transitions between flexible states, which underscore the heterogeneous yet organized conformational landscape of AICD. Although its structural ensemble is dominated by disorder, AICD preferentially populates specific conformational basins rather than uniformly sampling its energy landscape.

**Figure 12:**
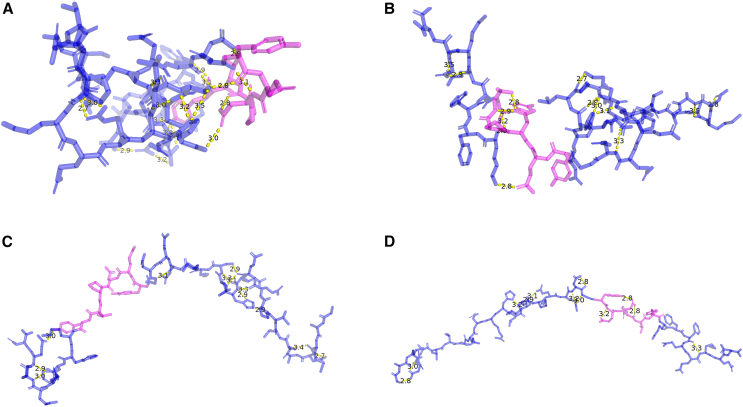
Snapshots of (*a*) Cluster 1, (*b*) Cluster 2, (*c*) Cluster 3, and (*d*) Cluster 4. The yellow dashed line represents the van der Waals’s distances, and the pink dashed line represents the dihedral angle distance. The YENPTY motif is highlighted in magenta.

Fig. 12 illustrates four representative structural clusters (a–d) obtained from the conformational analysis of a protein containing the YENPTY motif, shown in magenta. These clusters were derived from RMSD-based hierarchical clustering of conformations sampled throughout the MD trajectory, capturing distinct structural states adopted by the motif within the protein’s dynamic ensemble. Each cluster represents a distinct binding conformation, reflecting the dynamic interaction landscape of this conserved motif. van der Waals interactions, indicated by yellow dashed lines, depict close atomic contacts that may contribute to stabilizing the peptide within different binding pockets of the protein. π-interactions, shown as pink dashed lines, highlight potential aromatic or cation−π stacking events that could influence binding specificity and orientation. Cluster 1 (Fig. 12 *a*), which comprises approximately 71% of trajectory frames, adopts a relatively compact conformation with multiple van der Waals and π-interactions, which might correspond to a higher-affinity binding state. Cluster 2 (Fig. 12 *b*) (∼6% of frames) displays a more extended structure with fewer π-interactions, possibly representing a transient docking pose. Cluster 3 (Fig. 12 *c*) (∼3%) shows the motif in a bent conformation, which may indicate an alternative binding mode or regulatory state. Cluster 4 (Fig. 12 *d*) (∼1.5%) corresponds to a largely unbound or loosely associated configuration with minimal interactions, suggesting lower stability. These clusters collectively illustrate the potential structural adaptability of the YENPTY motif and its ability to engage in diverse interaction profiles depending on conformational context. However, further experimental validation is needed to confirm these models.

## Discussion

The structural and dynamic features of the AICD are intimately linked to its functional versatility in nuclear signaling and neurodegeneration. Despite its biological importance, AICD has remained structurally elusive due to its intrinsically disordered character. In this study, we provide a comprehensive, multimodal biophysical analysis of a 35-residue AICD segment containing the conserved YENPTY motif, an interaction hotspot relevant to AD and ALS pathology. Our combined use of MD simulations, SAXS, CD, and NMR data reveals that AICD samples a heterogeneous ensemble of conformations, characterized by dynamic compaction, transient secondary structure, and motif-specific flexibility.

Sequence-level analysis (Fig. 1) confirms a heterogeneous distribution of physicochemical properties, including a lack of contiguous hydrophobic clusters or segregated charge domains. This sequence composition is typical of intrinsically disordered proteins and suggests an intrinsic inability to fold into a single well-defined tertiary structure. The alternating pattern of hydrophobic and charged residues supports the potential formation of amphipathic, partially structured regions that may facilitate transient interactions without obligate folding.

Our MD simulations capture the evolution of the conformational ensemble, converging toward a quasi-equilibrium state with relatively stable RMSD values beyond ∼1.5μs (Fig. 2). The associated KDE reveals a unimodal distribution centered near 1.2 nm, indicating that the dominant ensemble is neither completely disordered nor tightly folded. Instead, AICD exists in a dynamic equilibrium between compact and extended conformers, corroborated by experimental SAXS and CD data (Figs. 3 and 8). The agreement between experimental and simulated SAXS profiles ($\chi^{2}$ = 1.162) and CD spectra (helical content 60%) validates the ensemble-averaged structural models. Notably, the Kratky and P(r) analyses confirm that AICD retains considerable flexibility, with only moderate compaction, and populates elongated states with intramolecular distances reaching 40 Å.

Structural heterogeneity is further captured by examining solvent exposure across the conformational ensemble. SASA analyses (Fig. 4) reveal that solvent accessibility scales with the radius of gyration, consistent with a shift from more compact to extended conformers. Residue-specific SASA values highlight terminal residues (e.g., G1, Q34, N35) as persistently exposed, whereas central hydrophobic residues (e.g., V7, F30, M33) undergo transient burial in compact states. These dynamics likely support context-dependent interactions or posttranslational modifications, especially given the high SASA variance observed for M33.

Contact maps and secondary structure profiles provide additional insight into transient folding behavior (Figs. 5 and 6). Lower $R_{g}$ ensembles exhibit short-range contacts and localized secondary structure, especially around the YENPTY motif, whereas extended ensembles lose these features. The dominance of loops, bends, and turns underscores AICD’s conformational plasticity, whereas scattered, low-probability α-helical and β-strand tendencies suggest the formation of transient structures. These motifs may act as nucleation points for interactions with partner proteins, particularly in signaling contexts.

Hydrogen bonding patterns (Fig. 7) reinforce the motif’s conformational sensitivity. In compact states, the YENPTY motif (Y20–Y25) participates in dense hydrogen bonding, indicative of partial ordering. As the structure expands, internal hydrogen bonding within the motif diminishes, although certain interactions (E21–T24) persist, suggesting residual structure even in more extended conformations. This compaction-dependent stabilization may represent a regulatory mechanism by which AICD modulates its binding capabilities.

The combined tICA and UMAP analyses (Figs. 10 and 11) reveal a rugged energy landscape populated by multiple metastable states. This is consistent with the functional paradigm of intrinsically disordered proteins, which often operate via conformational selection or induced fit mechanisms. The nonuniform occupancy of conformational space and the slow transitions observed in tICA imply that AICD transitions between states relevant for partner binding or posttranslational modification on biologically relevant timescales.

Finally, structural clustering of AICD bound to protein partners (Fig. 12) provides a potential model for interaction versatility. The YENPTY motif exhibits diverse binding poses, ranging from compact high-affinity conformations with abundant van der Waals and π-interactions to extended or loosely bound states. This versatility aligns with the motif’s known role as a modular docking platform in cellular signaling networks. Importantly, conformational-state-dependent solvent exposure and hydrogen bonding may regulate motif availability, providing a mechanistic basis for its dynamic functional repertoire.

## Conclusion

In this study, we employed an integrative structural approach combining MD simulations and SAXS to investigate the conformational behavior of the AICD. Our results reveal that AICD exists as a highly dynamic intrinsically disordered region, sampling a broad ensemble of conformations rather than adopting a single stable structure. Within this ensemble, we identified transient secondary structure elements that become more stabilized under compact conditions, particularly in the vicinity of the functionally critical YENPTY motif, which is known to be involved in protein-protein interactions and cellular signaling pathways. These observations provide a structural basis for understanding how AICD’s flexibility may contribute to its functional versatility.

Although our findings do not directly address the functional consequences of these dynamics, they establish a detailed biophysical framework that future studies can build upon. In particular, investigating how posttranslational modifications, such as phosphorylation, alter AICD’s conformational preferences and binding capabilities will be important for understanding regulatory mechanisms (ongoing study). Furthermore, applying time-resolved or in-cell experimental techniques could offer valuable insight into how AICD behaves within the complex environment of the neuron. Such studies will be critical for elucidating the role of AICD in neurobiology and for exploring how its dysregulation may contribute to disease, with potential implications for the development of targeted therapeutic strategies.

## Data and code availability

The datasets generated during and/or analyzed during the current study are available in the Amyloid Precursor Protein Intracellular Domain zenodo repository, https://doi.org/10.5281/zenodo.16420180.

## Acknowledgments

We acknowledge financial support from the Royal Society of Physiography in Lund, Sweden. The simulations were enabled by resources provided by the National Academic Infrastructure for Supercomputing in Sweden, NAISS, and partially funded by the 10.13039/501100004359Swedish Research Council through grant agreement no. 2022-06725 and resources provided by the Swedish National Infrastructure for Computing, SNIC, at the Center for Scientific and Technical Computing at Lund University, LUNARC, Sweden, partially funded by the 10.13039/501100004359Swedish Research Council through grant agreement no. 2018-05973. The 10.13039/501100001671European Synchrotron Radiation Facility, ESRF, provides beamtime MX-2673/2674. We sincerely thank our local contacts, Petra Pernot and Mark Tully, for their assistance.

## Author contributions

N.M.: conceptualization, methodology, validation, formal analysis, investigation, visualization, writing – original draft, review, and editing. M.S.: conceptualization, methodology, validation, resources, data curation, writing – original draft, review, editing, supervision, project administration, and funding acquisition.

## Declaration of interests

The authors declare no competing interests.

## Supporting material

Document S1. Figures S1–S9

Document S2. Article plus supporting material
