# Supplementary material for: Unraveling the conformational landscape of amyloid precursor protein intracellular domain
Source: Biophys J. 2025 Aug 14;124(21):3527–41. doi: 10.1016/j.bpj.2025.08.010 (PMC12709260; doi:10.1016/j.bpj.2025.08.010)
Supplement: Document S1. Figures S1–S9 [file mmc1.pdf]

Biophysical Journal, Volume *124*

Supplemental information

Unraveling the conformational landscape of amyloid precursor protein intracellular domain

Nabanita Mandal and Marie Skepö

Unraveling the Conformational Landscape of AICD: An Integrative SAXS and Molecular Dynamics Simulations Study

Nabanita Mandal† and Marie Skepö*,‡,¶

†*Division of Computational Chemistry, Department of Chemistry, Lund*

‡*Division of Computational Chemistry, Department of Chemistry, Science for Life Laboratory, Lund University, P.O. Box 124, SE-221 00, Lund, Sweden*

¶*NanoLund, Lund University, Box 118, 22100 Lund, Sweden*

E-mail: marie.skepo@compchem.lu.se

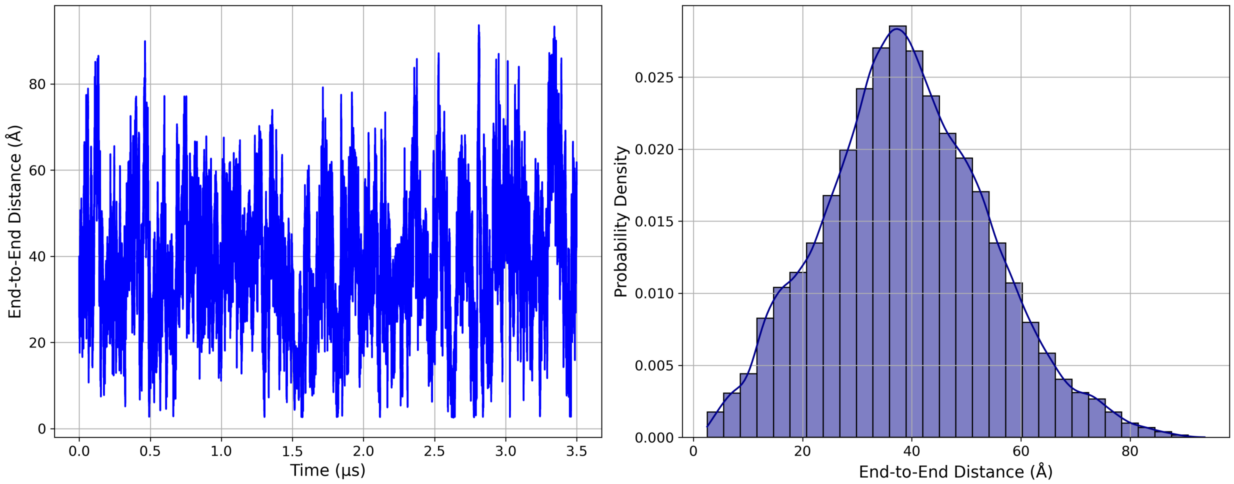

Figure S1: The end-to-end distance was measured from all-atom simulations conducted under 10mM salt conditions.

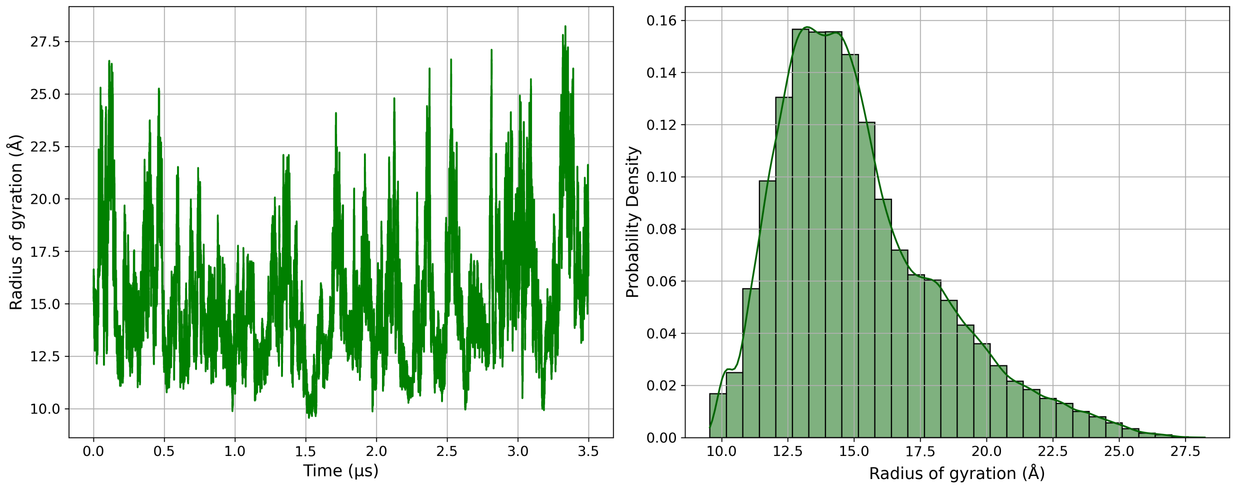

Figure S2: The radius of gyration R_g was calculated from all-atom simulations performed under 10mM salt concentration.

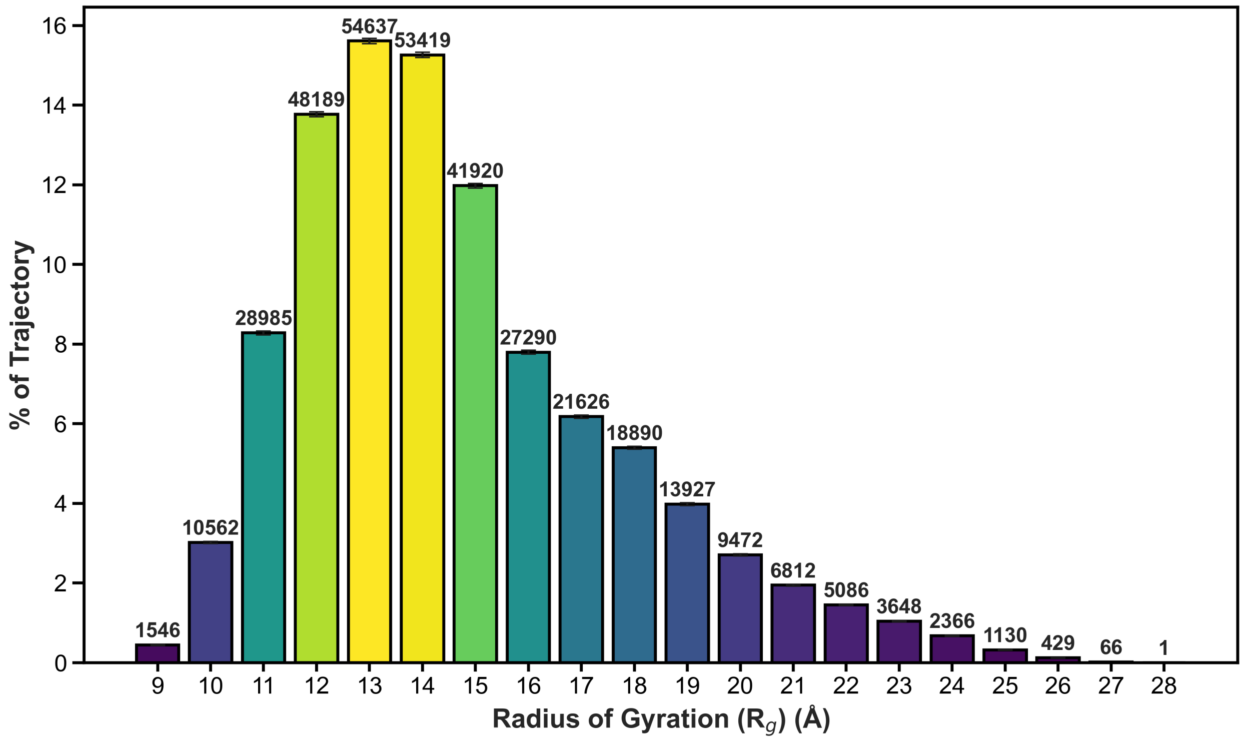

Figure S3: The decomposed conformational ensemble (CoE) is distributed across distinct radius of gyration (R_g) groups, with the number of frames within each group indicated above the bars. Error bars representing the standard deviation are shown in the Figure; however, they are relatively small, indicating low variability within each R_g group and suggesting that the observed distributions are robust. The Y-axis represents the percentage of the trajectory corresponding to each R_g group.

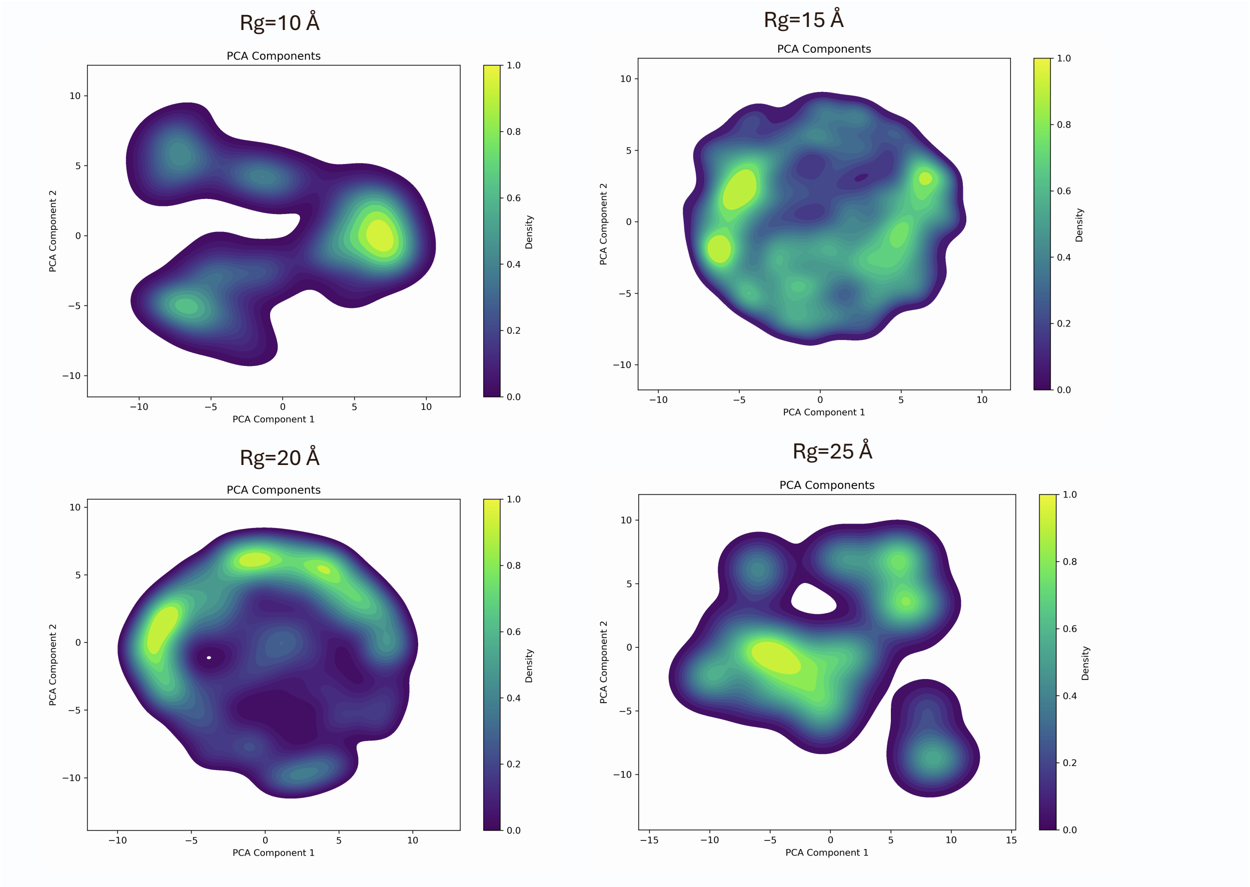

Figure S4: Principal Component Analysis (PCA) for different R_g groups, with the color scale representing the density displayed beside each plot.

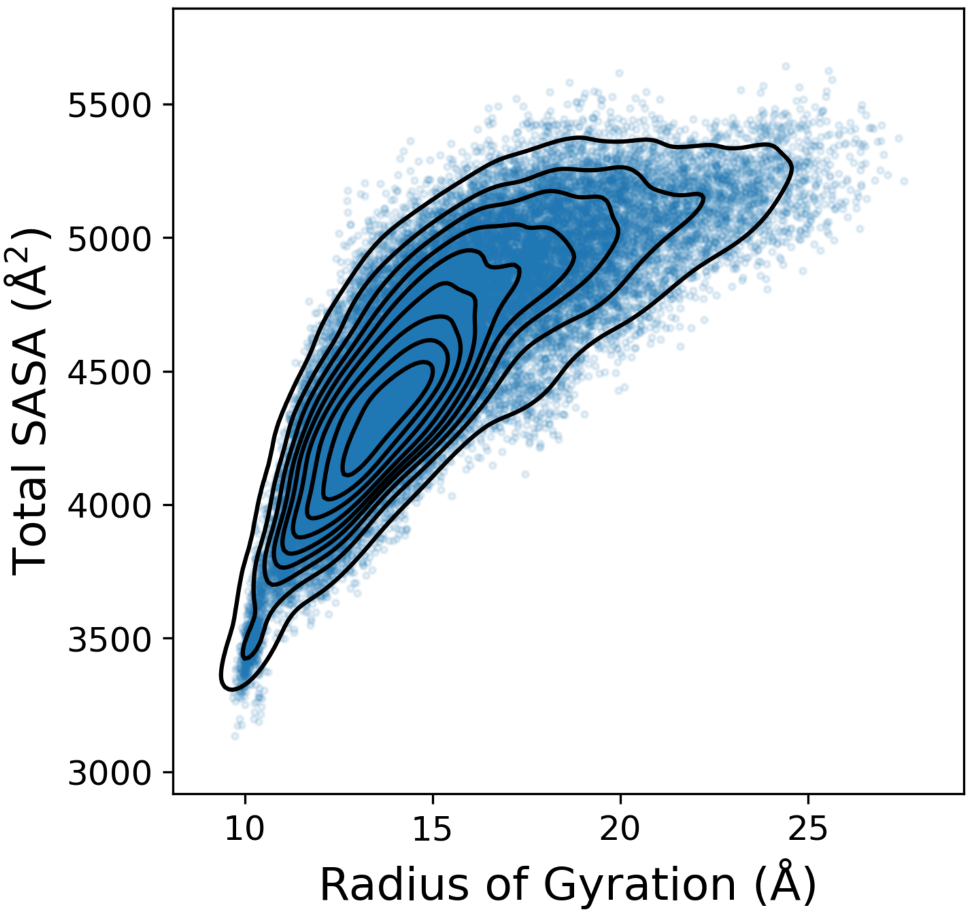

Figure S5: Plot of the radius of gyration (R_g) of the trajectory frames and their corresponding total solvent-accessible surface area (SASA).

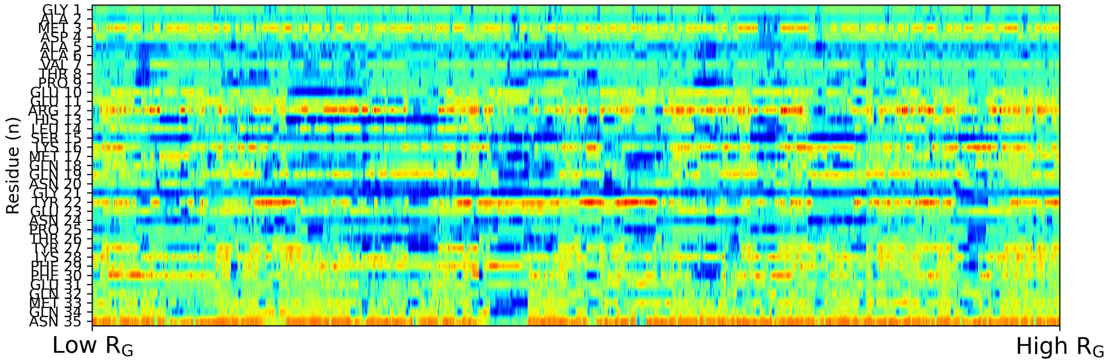

Figure S6: Heatmap of the total solvent-accessible surface area (SASA) by residues as a function of the increasing radius of gyration (R_g).

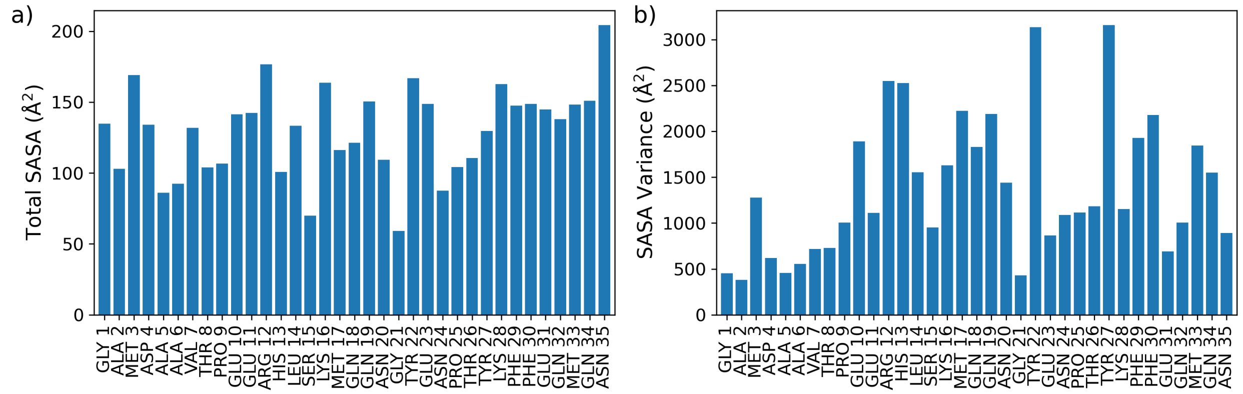

Figure S7: Bar plots of the solvent accessible surface area (SASA), shown as (a) the sums and (b) the variance across residues.

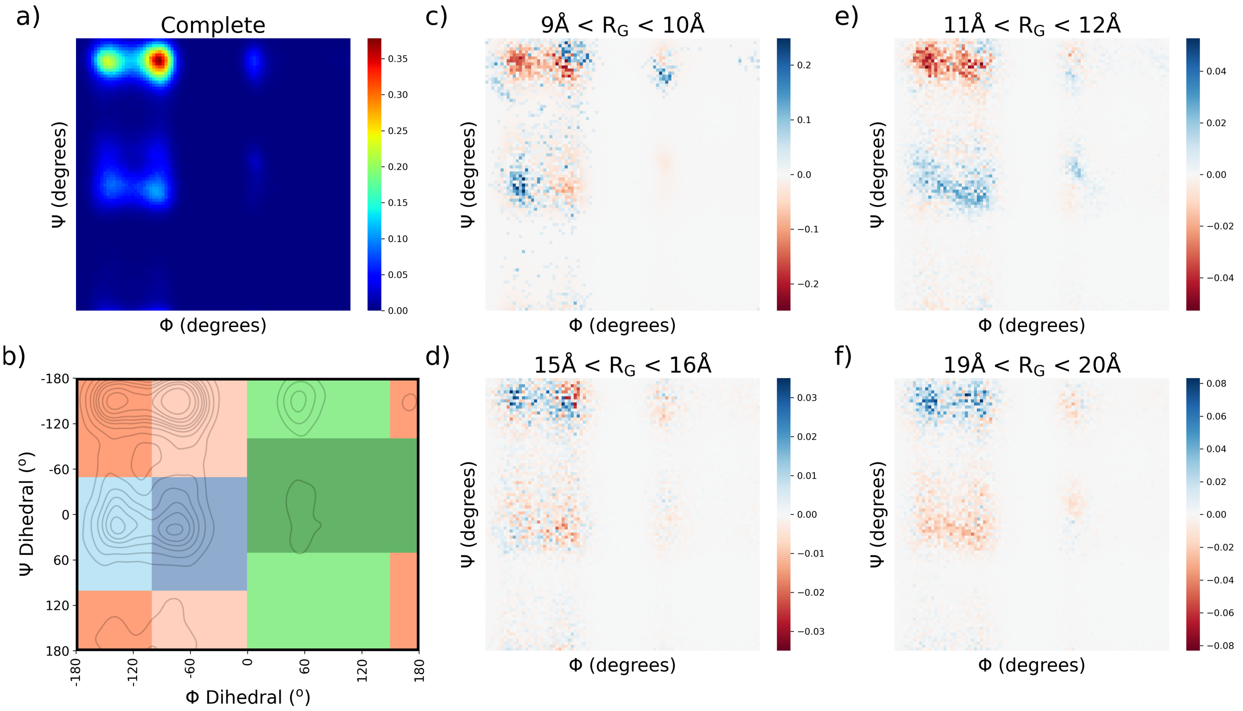

Figure S8: Integrated Ramachandran plots showing (a) the entire trajectory, (b) key secondary structural regions, and the differences in dihedral preferences for the groups of (c) 9 Å, (d) 11 Å, (e) 15 Å, and (f) 19 Å.

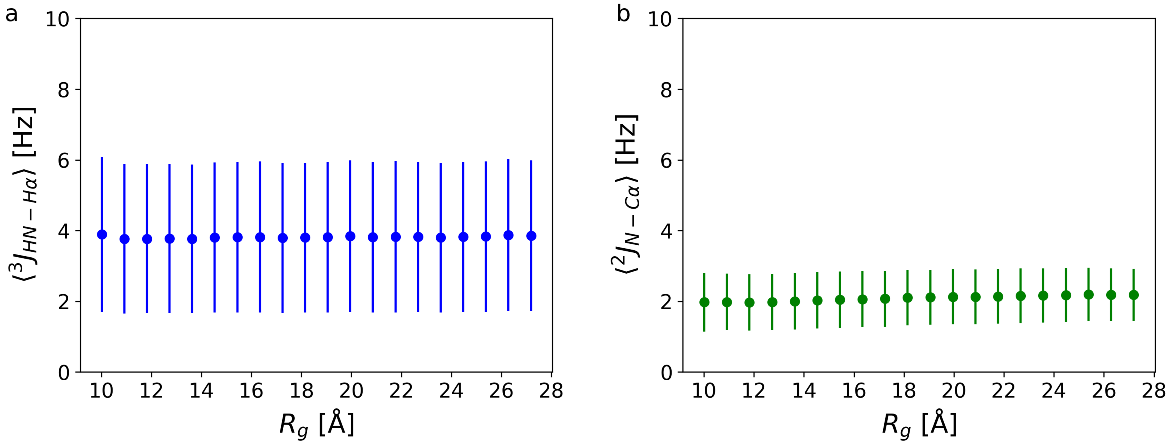

Figure S9: (a) J-coupling for the ϕ (Phi) angles and (b) for the ψ (Psi) angles as a function of radius of gyration (R_g). Error bars represent standard deviations, with each marker indicating the mean per residue.
